# Identification of Frameshift Variants in *POLH* Gene Causing Xeroderma Pigmentosum in Two Consanguineous Pakistani Families

**DOI:** 10.3390/genes13030543

**Published:** 2022-03-19

**Authors:** Ghazala Y. Zamani, Ranjha Khan, Noreen Karim, Zubair M. Ahmed, Muhammad Naeem

**Affiliations:** 1 Medical Genetics Research Laboratory, Department of Biotechnology, Quaid-i-Azam University, Islamabad 45320, Pakistan; ghazala.zamani@yahoo.com (G.Y.Z.); noreenmkd@gmail.com (N.K.); 2 Joint Centre for Human Reproduction and Genetics, Anhui Society for Cell Biology, School of Life Sciences, University of Science and Technology of China, Hefei 230052, China; rhanjha@mail.ustc.edu.cn; 3 Laboratory of Neurogenetics and Translational Research, University of Maryland School of Medicine, Baltimore, MD 21201, USA

**Keywords:** xeroderma pigmentosum, autosomal recessive, *POLH*, *REV3L*, *POLD2*, *REV7*, polymerase eta

## Abstract

Xeroderma pigmentosum (XP) is a rare autosomal recessive genetic disorder characterized by severe sensitivity of skin to sunlight and an increased risk of skin cancer. XP variant (XPV), a milder subtype, is caused by variants in the *POLH* gene. *POLH* encodes an error-prone DNA-polymerase eta (pol eta) which performs translesion synthesis past ultraviolet photoproducts. The current study documents the clinical and genetic investigations of two large consanguineous Pakistani families affected with XPV. In family 1, whole exome sequencing (WES) revealed a novel frameshift variant, c.1723dupG (p.(Val575Glyfs*4)), of *POLH*, which is predicted to cause frameshift and premature truncation of the encoded enzyme. Indeed, our ex vivo studies in HEK293T cells confirmed the truncation of the encoded protein due to the c.1723dupG variant. In family 2, Sanger sequencing of *POLH* exons, revealed a recurrent nonsense variant, c.437dupA (p.Tyr146*). POLH forms a hetero-tetrameric POLZ complex with REV3L, REV7, POLD2 and POLD3. Next, we performed in silico analysis of *POLH* and other POLZ complex genes expression in publicly available single cell mRNAseq datasets from adult human healthy and aging skin. We found overlapping expression of *POLH, REV3L* and *POLD2* in multiple cell types including differentiated and undifferentiated keratinocytes, pericytes and melanocytes in healthy skin. However, in aging human skin, *POLH* expression is reduced in compare to its POLZ complex partners. Insights from our study will facilitate counseling regarding the molecular and phenotypic landscape of *POLH*-related XPV.

## 1. Introduction

The genomes of the living cells are continuously exposed to attack by different exogenous and endogenous toxic agents. To eliminate errors and to reserve the biological information of the DNA, different DNA repair pathways exist within the cells to maintain its integrity. Defective DNA is repaired by two different procedures, i.e., nucleotide excision repair (NER) and post replication repair (PRR), [1]. NER is further divided into two subtypes, i.e., global genome repair (GGR), in which damages to the DNA of the entire genome are repaired, and transcription-coupled NER repair (TC-NER), in which damages on the transcribed strand of actively transcribed regions of the DNA are repaired [2]. However, the failure of these repair systems can lead to different DNA lesions, which cause many human disorders [3]. Xeroderma pigmentosum (XP) is a rare, clinically heterogeneous, autosomal recessive genetic disorder defined by mild to severe sensitivity of the skin to sunlight resulting in sunburn, lentigines at exposed skin areas and early skin aging, with increased incidence of malignancies [4]. Photophobia has been reported in a few cases, while some patients also have neurological problems, causing progressive neurological disabilities and early death. XP patients have a high incidence of skin cancers and tumors on the exposed skin areas as their cells are not able to repair DNA damage induced by UV radiation [5]. Clinical diagnosis is established by cellular tests for altered DNA repair [6].

XP affected individuals have defects in the repair of DNA damage induced by UV and other DNA damaging agents. Variants in eight genes associated with eight complementation groups (XP-A (*ERCC1*), XP-B (*ERCC3*), XP-C (*XPC*), XP-D (*ERCC2*), XP-E (*DDB2*), XP-F (*ERCC4*), XP-G (*ERCC5*) and XP-V (*POLH*)) are known to cause XP. Genetically, XP can also be classified as classical XP (XPA to XPG) and XP variant (XPV) [7]. The *POLH* (XPV) encodes DNA polymerase η, a member of the Y-DNA polymerase family, acts as a translesional DNA polymerase and is essential for the replication of unrepaired DNA damage induced by UV. XPV cells have normal GGR and TC-NER but are defective in post replication repair due to which cells can replicate DNA lesions unrepaired by NER [2,8]. The defective XPV cells are retrained to use a more error-prone polymerases for the replication of the damaged DNA template, which increases the chances of mutations much more than the normal cells. The patients show photosensitivity, altered skin pigmentation on the exposed areas and increased risk of skin cancer compared to the general population [9]. Currently, there is no complete treatment of XP and, therefore, photo protection, proper management, understanding of the associated neurological problems in some cases and timely diagnosis with general awareness about the disorder in the affected families are very important and can improve the living style of the patients [6].

In the current study, we ascertained two consanguineous Pakistani families affected with XP, and through WES and Sanger sequencing, identified one novel and one recurrent likely pathogenic *POLH* variant. We also functionally evaluate the impact of the novel variant through expression studies in heterologous cells.

## 2. Methods

### 2.1. Participants

The study was approved by the Institutional Review Board of Quaid-i-Azam University Islamabad. Two extended consanguineous Pakistani families suffering from xeroderma pigmentosum were recruited in the current study. Informed consents were obtained from all participating family members. Peripheral blood samples were collected to extract genomic DNA by using QIAamp DNA blood Mini Kit (Qiagen, Hilden, Germany) according to the provided instructions.

### 2.2. Whole Exome Sequencing (WES) and Bioinformatic Analysis

WES was performed on the genomic DNA of two affected individuals of family 1 (IV-1, IV-4) and one parent of each of them (III-5, III-9). Extracted DNA was subjected to fragmentation and an indexed individual library was prepared for each sample using Agilent SureSelect Human All exon v5 kit. Sequencing was performed on a Hiseq2000 platform (Illumina). Sequencing reads (qseq format) were aligned to the human genome (GRCh37/hg19) using Novoalign V2.07.13 (http://www.novocraft.com/index.html accessed on 22 May 2021) with default parameters. By using SAM tools (http://samtools.sourceforge.net/ accessed on 23 May 2021), the SAM file of each sample was converted to a BAM file, sorted and merged. PCR duplicates were removed using Picard (http://picard.sourceforge.net/ accessed on 25 May 2021). Files were further processed using Genome Analysis Tool Kit from the Broad Institute (http://www.broadinstitute.org/gatk/ accessed on 15 June 2021). All BAM files were locally realigned using indel realigner. Both single nucleotide variants (SNVs) and small INDELs (insertions and deletions) within the captured coding exonic intervals were called using GATK’s Unified Genotyper. From the genomic DNA of four samples of family 1 (IV1, IV:4, III:5 and III:9), an average of 6.475 GB DNA sequences were generated. We used tiered filtration strategy (Supplementary Figure S1) to narrow down the candidate disease-causing variants for segregation analysis. Several in silico algorithms, including PolyPhen2 HDIV, Polyphen2 Hvar, MutationTaster, GERP^++^, SiPhy, fathmm-MKL, SIFT, MutationAssesor, FATHM, Meta-SVM, MetaLR and Provean, were used to predict the impact of identified variants on the encoded proteins.

### 2.3. Primers Design, Polymerase Chain Reaction (PCR) and Sanger Sequencing

For segregation analysis and Sanger sequencing of *POLH* gene primers were designed through Primer3 software. PCR products were generated using high fidelity PrimeSTAR^®^ Max DNA Polymerase (Takara, Shiga, Japan), purified with GeneJET Purification Kit (Thermo Fisher Scientific, Waltham, MA, USA) and Sanger sequenced using Beckman Coulter CEQ8800 (Beckman Coulter, Pasadena, CA, USA). Variations in sequences were then analyzed by using BioEdit sequence alignment editor version 6.0.7.

### 2.4. Protein Modeling and mRNA Expression Studies

Wild type and mutant POLH protein 3-Dimensional (3D) structures were generated through Phyre2 program, and visualized by Chimera. UCSC cell browser (https://cells.ucsc.edu/ accessed on 12 July 2021) was used to obtain human healthy (*n* = 5) and aging (*n* = 5) skin single cell RNA seq data plots.

### 2.5. POLH Constructs, Transfection, and Protein Expression

The wild type *POLH* (NM_006502) was amplified from HEK 293T cell lines, and sub-cloned into pEGFP-N1 vector by using ClonExpress II one step cloning kit (Vazyme Biotech, China). Human embryonic kidney (HEK) 293T cell lines acquired from ATCC (cat# CRL-1573) were cultured in Dulbecco’s Modified Eagle Medium (DMEM) containing 10% fetal bovine serum (FBS) with addition of penicillin and streptomycin. The wild type and mutant plasmids (250 ng) were transfected into the cultured HEK293T cell lines by using Lipofectamine 3000 (Thermo Fisher Scientific, Waltham, MA, USA). Expression of the proteins were observed after 48 h of transfection under Nikon ECLIPSE 80i fluorescence microscope(Nikon Corporation, Tokyo, Japan). Western blot analysis was performed to confirm the expression of exogenous POLH proteins and to evaluate the impact of identified novel variant.

## 3. Results

**Figure 1:**
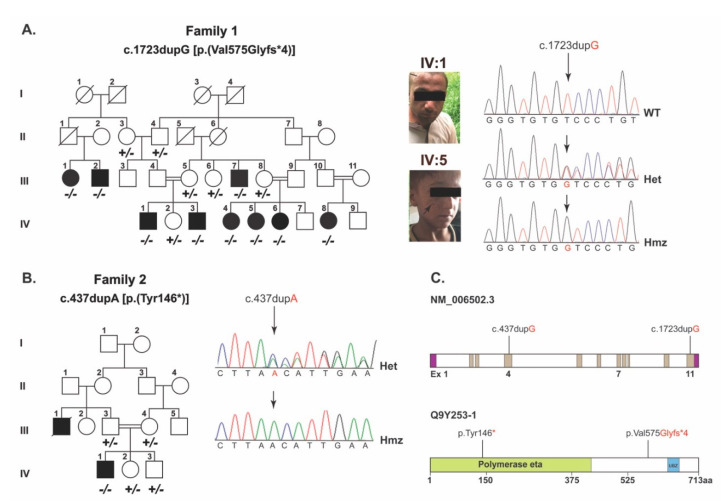
**Pedigrees of the two unrelated families affected with xeroderma pigmentosum.** (**A**) Pedigree of family 1 along with their photographs and chromatograms generated with Sanger sequencing data. All the patients were offspring of first-cousin marriages. The patients have lentigines on the exposed parts of the body, especially face and neck. (**B**) Family 2 pedigree and sequencing chromatograms. Filled symbols represent affected individuals, double lines represent consanguineous marriages. Generation numbers are shown as I, II, III and IV. (**C**) Schematic representation of *POLH* gene and protein structure. The identified variants (c.437dup A and c.1723dupG) were present in *POLH* exon 4 and 11, respectively. Polymerase eta domain is involved in translesion synthesis; however, UBZ (Ubiquitin-Binding Zinc Finger) is necessary for the polymerase engagement to the impeded replication machinery in translesion.

As part of our ongoing efforts on understanding the genetic basis of skin disorders in humans, two consanguineous families segregating XP in autosomal recessive fashion were recruited from the Punjab province of Pakistan (Figure 1a,b). Family 1 comprised nine affected members with proneness to sunburn and early development of an atypical melanocytic lesion. Family 2 comprised three affected members with sunburning and lentigines on sun-exposed body parts (Figure 1a, arrows) and had mild photophobia.

**Figure 2:**
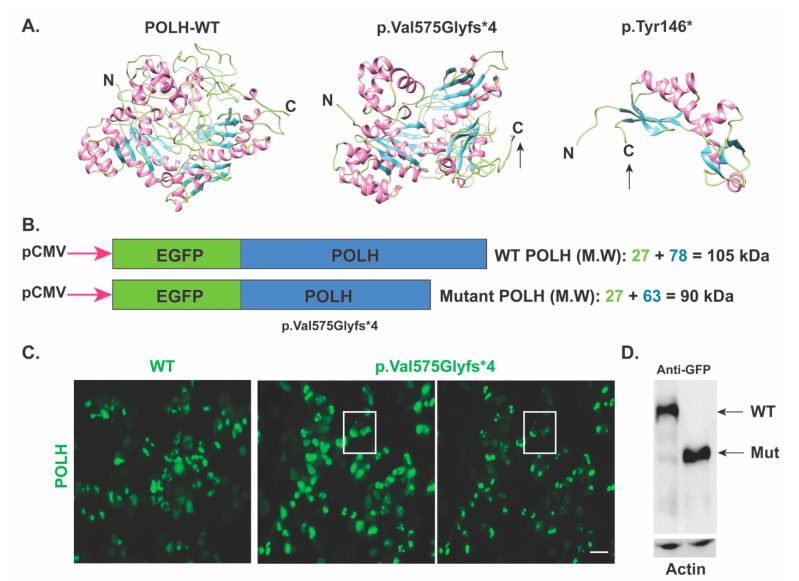
Wild type and mutant POLH expression in HEK293T cell lines. (**A**) POLH protein 3D structures. Protein helix, sheets and coils are shown in pink, blue and yellow colors, respectively. N and C terminals are labeled; however, truncation is marked by using arrow. (**B**) Graphical representation of POLH WT and mutant protein. Plasmid constructs were transfected in HEK293T cells. (**C**) Wild type and mutant POLH expression in HEK293T cell lines. The mutant (p.Val575Glyfs*4) protein displayed punctate expression in cells. (**D**) Western blot image of POLH WT and mutant protein using GFP and POLH antibody.

In family 1, employing WES and the subsequent filtration criteria (Supplementary Figure S1) identified a frameshift variant, (c.1723dupG; p.(Val575Glyfs*4)), in *POLH*. Sanger sequencing of DNA samples from all the participants confirmed the co-segregation of c.1723dupG variant with XP in family 1 (Figure 1a,c). This variant is not found in the gnomAD, 1000 genomes and ESP6500 databases, and is predicted to cause reading frameshift and premature truncation of the encoded POLH protein at the polymerase eta domain (Figure 1c and Figure 2a).

To decipher the genetic cause of family 2, we used the candidate gene approach and performed Sanger sequencing of the exonic sequences and adjacent splice junctions of *POLH.* We identified a previously reported c.437dupA (p.Tyr146*) variant in exon 4 (Figure 1b), which co-segregated with XP in family 2. The variant was not found in the 100 control Pakistani samples or in our in-house exome sequences database of an unrelated normal Pakistani population (*n* = 50). However, in the gnomAD database, the c.437dupA variant has a minor allele frequency of 8.239 × 10^−6^ with no reported homozygotes. The c.437dupA variant is predicted to truncate the POLH at the low complexity domain region (Figure 1c and Figure 2a).

Next, to confirm the predicted impact of the novel c.1723dupG; (p.Val575Glyfs*4) variant, the *POLH* wild type and mutant cDNA incorporated in pEGFP-N1 vector (for fluorescence visualization) were transfected into HEK293T cells. Based on the predicted impact, we anticipated that the *POLH* harboring p.Val575Glyfs*4 allele would yield a truncated protein of around ~90 kDa (GFP 27 kDa + 63 kDa truncated protein) as compared to the 101kDa wild type protein (Figure 2b). Indeed, Western blotting using anti-GFP antibodies confirmed, at least ex-vivo, the truncation of POLH due to c.1723dupG; (p.Val575Glyfs*4) variant (Figure 2c). Furthermore, when analyzing the expression in HEK293T cells using anti-POLH antibodies, as compared to diffuse cytoplasmic localization of wild type protein, the truncated POLH due to the p.Val575Glyfs*4 variant had a punctate pattern (Figure 2d), further supporting the damaging impact of the identified variant on the encoded POLH. These results suggest that even though the over-expressed mRNA harboring c.1723dupG variant escaped nonsense-mediated mRNA decay, the mutant protein lacks a portion of the C-terminal, which might render the protein non-functional; therefore leading to the disease phenotype in the studied family1.

**Figure 3:**
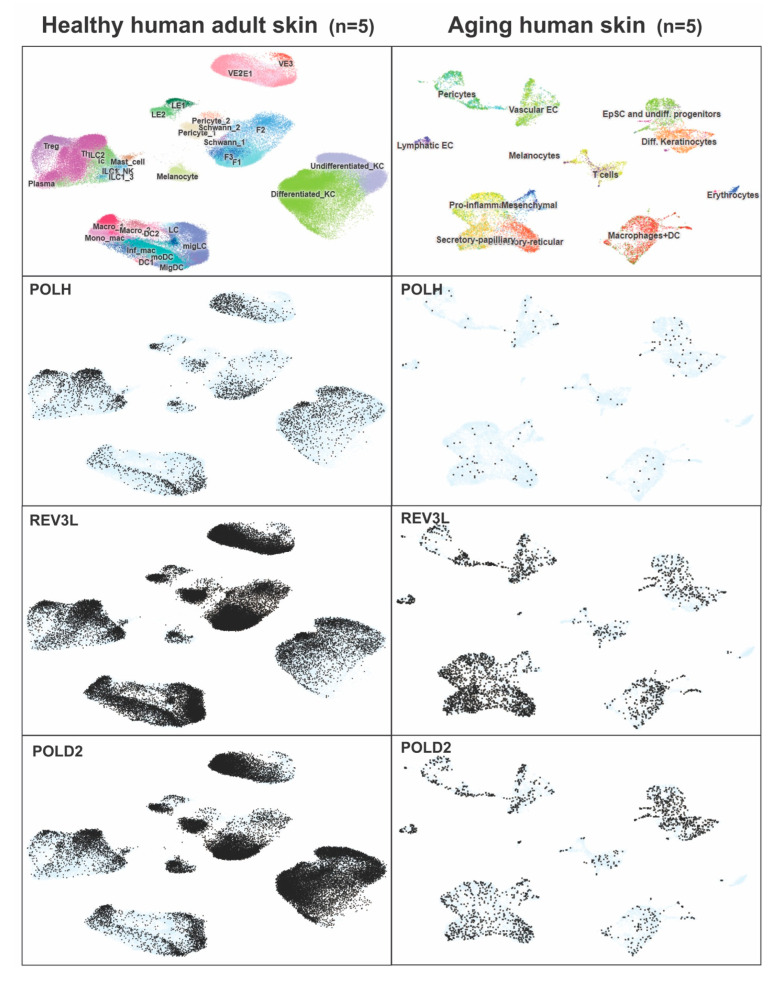
Single cell RNA seq data showing expression of *POLH* in human healthy and aging skin. This dataset was generated from 500,000 cumulative single cells (healthy) and 5000 fibroblasts of aging human skin. Data are plotted using UMAP (Uniform Manifold Approximation and Projection for Dimension Reduction). Gene expression is highlighted in black dots; however, light blue shade represents no expression.

POLH is known to make a hetero-tetrameric complex with Pol zeta subunits (REV3L, REV7, POLD2 and POLD3) [10]. Therefore, we investigated the expression of POLZ complex proteins, and their cell-type specific regulation in the healthy as well as aging human skin cells. For this study, single cell mRNAseq data were obtained from publicly available databases. Among the Pol zeta subunits, we observed overlapping expression of *POLH, REV3L* and *POLD2* in multiple cell types including differentiated and undifferentiated keratinocytes, pericytes and melanocytes in healthy skin (Figure 3). However, in aging human skin, *POLH* expression is reduced in comparison to its POLZ complex partners (Figure 3).

## 4. Discussion

The DNA polymerase eta, a 713 amino acids long protein encoded by *POLH*, has a highly conserved amino terminal throughout the members of Y-family polymerases, which contains the active site responsible for the polymerase activity, while the C-terminal of the protein (~120 amino acids) is vital for the regulation of nuclear localization and for gathering into replication forks after UV radiation [11]. The function of the central 240 amino acids is not yet known. Thus far, ninety-two variants in *POLH* associated with XPV type have been reported scattered throughout the gene including missense, nonsense, indels and splice site variations (http://www.hgmd.cf.ac.uk; accessed 01 October 2021). In the current study, a novel frameshift variant c.1723dupG (p.Val575Glyfs*4) in exon 11 of the *POLH* gene was identified in family 1, which is predicted to truncate the carboxy end of the encoded enzyme, in the event mutant mRNA escapes the nonsense mediated decay pathway. The C-terminal part of the enzyme has an essential role in the nuclear localization and accumulation of the protein at the replication forks of damaged DNA [11]. Indeed, our ex vivo studies revealed impaired trafficking of the encoded enzyme harboring the p.Val575Glyfs*4 variant. Extrapolating from these ex vivo studies, it is likely that in affected individuals, the mutant pol eta type identified here would not be able to perform its function in the DNA repair due to impaired trafficking.

In family 2, we found a c.437dupA (p.Tyr146*) variant in the 4th exon of *POLH* previously reported in two patients of different origins. [9,12]. This variant lies in the conserved region of the active catalytic domain of the polymerase eta. However, there might not be any functional mRNA due to nonsense-mediated mRNA decay, leading to an absence of the active polymerase eta protein.

XP has a wide range of symptom severity and clinical features among the eight complementation groups (XP-A to XP-G and XP-V). The phenotype of XPV patients, which is observed in 7% of all XP patients worldwide is generally described as milder based on their cellular sensitivity to UVR and lack of neurological abnormalities, but they tend to develop more skin cancers than other XP groups [13,14]. However, the recurrent variant c.437dupA (p.Tyr146*) identified in the current study was previously reported in an African black-skinned patient who did not develop skin cancer at all (diagnosed at the age of 42) despite important sun exposure without photoprotection (lived in Congo at the equator level). This is probably linked to her black skin, which is very protective against tumors, even in an XP background [9].

Recently, a 16-year-old girl affected with XPV was identified with two heterozygous truncating POLH mutations in trans (a deletion in exon 6 and a frameshift mutation in exon 11). She was born to non-consanguineous parents and presented with uncommon features including multiple erythematous, nodular skin lesions and substantial actinic keratoses of the face and upper chest. Skin biopsies confirmed multiple basal cell and invasive squamous cell carcinomas. She developed early symptoms (at 16 years) as compared to other XPV patients who typically show a late median onset of 24 years [14].

The genotype–phenotype relationship in XPV patients remains uncertain. This may be attributed to the fact that the severity of clinical features is not determined by the type and localization of the mutation alone but factors such as the intensity of sun exposure, age, living conditions of the patients and other genetic determinants, e.g., protective polymorphisms might also be involved [15]. Founder mutations in the *POLH* gene have been reported in some populations such as Japanese, Korean and Tunisian. Therefore, 87% of the Japanese XPV patients shared one of the four founder mutations described in Japan [16].

Our scRNA expression analysis in healthy human skin supports the presence of a POLH-mediated Pol zeta hetero-tetrameric complex in multiple cell types including differentiated and undifferentiated keratinocytes, pericytes and melanocytes. However, in aging human skin, *POLH* expression is reduced in comparison to its POLZ complex partners, potentially indicating the reasons behind the gradual loss of the skin’s ability to protect against the different exogenous and endogenous toxic agents with aging.

## Supplementary Materials

The following supporting information can be downloaded at: https://www.mdpi.com/article/10.3390/genes13030543/s1, Figure S1: Number of candidate variants at successive steps of filtrating WES data to identify disease-causing variant.
