# Supplementary figures and images for: Identification of Frameshift Variants in POLH Gene Causing Xeroderma Pigmentosum in Two Consanguineous Pakistani Families
Source: Genes (Basel). 2022 Mar 19;13(3):543. doi: 10.3390/genes13030543 (PMC8955859; doi:10.3390/genes13030543)

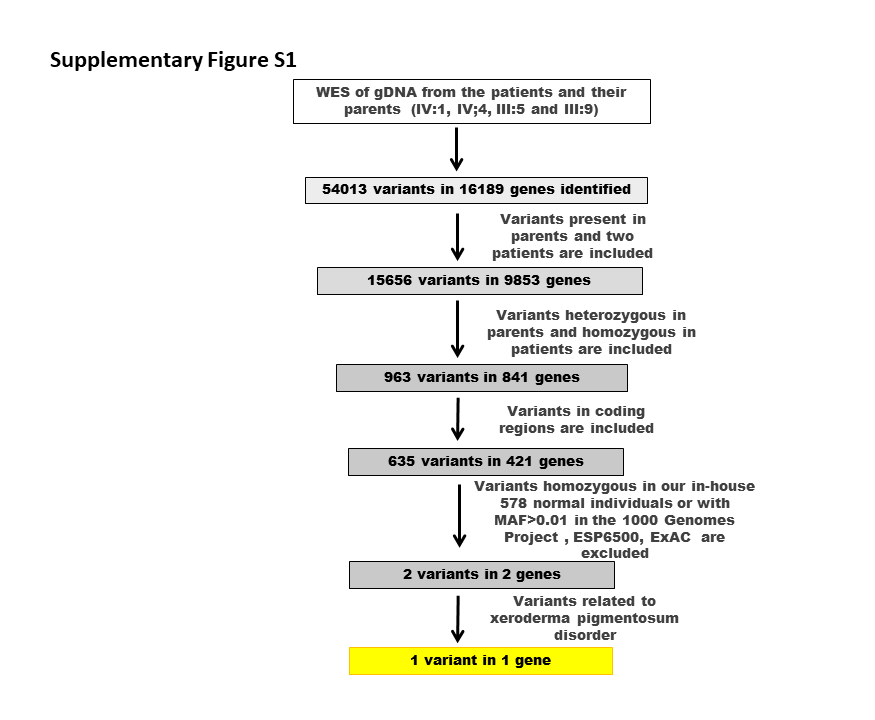

Supplement: Supplementary file 1 [file genes-13-00543-s001.zip › genes-1624371-supplementary.TIF]
